# Physical Properties of Glass-Fibre-Reinforced Polymer Filled with Alumina Trihydrate and Calcium Carbonate

**DOI:** 10.3390/polym14122464

**Published:** 2022-06-17

**Authors:** Djoko Setyanto, Yohanes Agus Jayatun, Prita Dewi Basoeki, Anthon De Fretes

**Affiliations:** 1 Mechanical Engineering Department, Faculty of Engineering, Atma Jaya Catholic University of Indonesia, Jakarta 12930, Indonesia; pdbasoeki@atmajaya.ac.id (P.D.B.); anthon.defretes@atmajaya.ac.id (A.D.F.); 2 Mechanical Engineering Department, Faculty of Industrial Technology, Institut Teknologi Nasional Yogyakarta, Yogyakarta 55281, Indonesia; jayatun@itny.ac.id

**Keywords:** GFRP, unsaturated polyester, calcium carbonate, alumina trihydrate, gutter

## Abstract

Gutters made of glass-fibre-reinforced polymer (GFRP) are usually produced with a three-millimetre thickness. The fillers are mixed into unsaturated polyester (UP) resin, which is intended to make the composite material more affordable. This study aims to examine the effects of the addition of alumina trihydrate (ATH), calcium carbonate (CC), and a mixture of ATH and CC of 15 and 30 parts per hundredweight of resins (PHR) on the material properties of the three-millimetre-thick three-layered GFRP composites. The properties observed included physical properties, namely, specific gravity and water absorption, chemical properties such as burning rate, and mechanical properties such as hardness, flexural strength, and toughness. The effects of the fillers on the voids and interfacial bond between the reinforcing fibre and matrix were analysed using the flexural fracture observation through scanning electron microscopy (SEM). The results showed that the addition of fillers into the UP resin led to an increase in the density, hardness, flexural strength, modulus of elasticity, and toughness but a decrease in water absorption and burning rate in a horizontal position. This information can be helpful for manufacturers of gutters made of GFRP in selecting the appropriate constituent materials while considering the technical and economic properties.

## 1. Introduction

Gutters in various buildings currently still predominantly use conventional steel materials. Problems that usually occur in gutters are about the durability and function of gutters due to the corrosion of these steel materials. Therefore, steel plates for gutters are usually provided with a corrosion protective coating. Various methods have been applied to protect steel materials from degradation due to corrosion, be it coatings with polymers or galvanising coatings of zinc or zinc-aluminium alloys and coatings with polymers [1,2]. The protective layer of the steel gutter of polymer material can be worked in the field by painting or spraying/rubbing the polymer material. Likewise, hot galvanization mainly carries zinc or zinc–aluminium protective coatings on steel coils in factories [3,4]. Researchers are still trying to develop techniques for coating corrosion protection with metal alloys or relatively inexpensive polymer materials: for instance, using polyaniline coating with the deposition electrophoresis technique [5]. Steel corrosion cannot be stopped entirely but is only inhibited by a protective layer. Modern composite-based materials resistant to corrosion are a promising alternative to replace conventional materials.

Construction materials in civil engineering have commonly relied on modern materials, one of which is the glass-fibre-reinforced polymer (GFRP) composite material [6,7,8,9,10,11]. One of the products made of GFRP is gutters, which are usually manufactured with a thickness of three millimetres and a length of six metres [12]. During the installation, the connection between gutters uses the same GFRP materials, resulting in no visible connection. These GFRP materials are often preferable to conventional steel materials because the former is lighter, easier to fabricate, more corrosion-resistant, and easier to repair in case of damage [13,14,15]. These advantages also apply to gutters made from GFRP materials compared to those made from steel plates.

GFRP materials include polymer matrix composite (PMC) materials, which has a higher specific strength and stiffness compared to steel [16]. They are also easier to make and do maintenance on. Apart from being free from corrosion problems, other technical properties, especially strength and stiffness, can be adjusted according to design requirements by selecting the fibre type, orientation, and fibre volume fraction [17]. The type, size, length, and orientation of the fibre and the fibre volume fraction will determine the engineering properties of the PMC design based on the Rule of Mixture.

Technical properties of gutters are essential for manufacturers to consider. Gutters are supported by bracket structures, with the distance between brackets usually half a metre. In addition to their primary function of collecting rainwater flowing on the roofs and transferring it into the pipe and channel underneath, gutters are useful for workers to step on when they are cleaning or repairing the roofs or gutters. Given that, the GFRP materials for gutters should have sufficient flexural strength and impact strength, not to mention the behaviour of water/moisture absorption properties.

Besides the technical aspect, the investment of gutters is also made as economical as possible so that they are widely affordable for the user community. To meet this economic factor, orthophthalic unsaturated polyester (UP) resin and E-glass reinforcing fibre can be selected as the composite constituent materials [7,8,18]. Further, fillers with added resin can also provide more economical value [19]. In Indonesia currently, the prices of the primary constituent materials of GFRP are E-glass roving/chopped 1.2 to 2.0 USD, UP resin 2.2 to 2.3 USD, alumina trihydrate filler 0.6 to 0.9 USD, and calcium carbonate filler 0.05 to 0.06 USD. In addition to reducing costs, the addition of fillers can also improve composite properties, resulting in an increase in the properties of the composite material. However, it is worth noting that an excessive amount of fillers will damage the material properties, potentially causing agglomeration, wherein the filler does not mix homogeneously with the resin. This will consequently cause the composite material to exacerbate in its quality and become brittle [19,20,21,22,23,24,25,26,27,28,29,30].

Among many fillers used commercially by manufacturers in the mixture of UP resins are alumina trihydrate (ATH) and calcium carbonate (CC). Previous studies have been conducted on GFRP and polymer matrix composites that use ATH or CC fillers. They show how the tensile strength, flexural strength, impact strength, and water absorption of composites with these fillers were reported to change, vis-à-vis composites with pure resins without fillers. Zhao et al. [21] investigated the effect of Al(OH)_3_ filler on the mechanical properties of unsaturated polyester matrix composites. The use of fillers to 10 PHR could improve the mechanical property of the composite—in this case, its tensile strength—but the addition of fillers beyond 10 PHR could damage the property. The modulus of elasticity increased almost linearly for fillers up to 40 PHR, after which it decreased. Likewise, the composite hardness increased for the use of fillers up to 30 PHR. Petersen et al. [22], who investigated GFRP composites with UP resin and ATH 25 PHR and 50 PHR fillers, also reported that the use of ATH 25 PHR filler could result in increasing flexural strength. Another study by Zainudin et al. [23] demonstrated a similar finding that the addition of ATH filler up to 40 PHR into the UP resin in GFRP composites could improve the modulus of elasticity. UP resin composite with ATH filler up to 7.5 PHR with kenaf fibre showed improved properties. Tensile strength, flexural strength, and impact strength increased, while water absorption decreased [24].

Another study by Borkar et al. [19] examined the effect of silica and calcium carbonate fillers in UP resins on the properties of woven glass fibre composites to further observe the amount of loss of properties due to the replacement of expensive resins with cheap fillers. Composites filled with silica and calcium carbonate fillers of 25 PHR and 50 PHR resulted in lower tensile strength but higher flexural strength. Other researchers studied HDPE composites filled with calcium carbonate (CaCO_3_) filler of up to 30 PHR as well as kenaf and rice husk [25]. The flexural strength of the hybrid composite increased with the addition of CaCO_3_ filler up to 20 PHR and dropped when the addition exceeded 20 PHR. Sravani et al. [26] reported their research on glass fibre/epoxy composites using fillers CaCO_3_ and Al_2_O_3_ with variations of 0, 5, and 10 PHR. They revealed significant improvements in the mechanical properties, namely, impact strength and hardness. Other researchers [27,28,29] also had similar findings that adding CaCO_3_ filler with the amount of 5 to 10 PHR increased tensile strength. Moreover, the flexural strength, modulus of elasticity, impact strength, and hardness could increase with the addition of fillers of up to 25 PHR.

The GFRP composite for gutters with a thickness of three millimetres typically consists of three layers of reinforcement: two layers of chopped strand mat of “450 g/m^2^” in the outer layers and one layer of woven roving of “800 g/m^2^” in the middle. Many researchers have conducted studies related to the engineering properties of GFRP composite materials. However, the specifics of GFRP as gutter material mentioned above are not well known. It is hitherto still unknown how the engineering properties of the GFRP composites will be with the three layers of the reinforcements based on a matrix of orthophthalic UP (Ortho-UP) resin as gutter materials with various fillers of ATH only, CC only, or a combination of ATH and CC. Therefore, it is necessary to research the effect of GFRP with this composition on its physical, chemical, and mechanical properties. In particular, the present study focused on density, water absorption, fire resistance, hardness, flexural strength, impact strength, and fracture morphology. This information can be beneficial to ensure good engineering properties and the reasonable price of the GFRP gutters.

## 2. Materials and Methods

### 2.1. Materials

The GFRP composite samples consisted of the following materials: orthophthalic unsaturated polyester resin (Ortho-UP), E-glass chopped strand mat of “450 g/m^2^” (CSM450), E-glass woven roving of “800 g/m^2^” (WR800), alumina trihydrate (ATH), and calcium carbonate (CC). The matrix used Ortho-UP SHCP 3316QN resin (PT SHCP Indonesia, Surabaya, Indonesia). The composite curing process used one percentage volume of resin as the initiator of methyl ethyl ketone peroxide (MEKP) MEPOXE M (PT Kawaguchi Kimia, Jakarta, Indonesia). The fillers were CC (Jia Dah Chemical Industrial Co. Ltd., Tainan, Taiwan) and ATH (Hindalco Industries Ltd., Kolkata, India). The composite reinforcement used three reinforcements: CSM450 + WR800 + CSM450 (PT Makmur Fantawijaya Chemical Industries, Jakarta, Indonesia).

The diameter of E-glass fibre is 15 ± 2 microns in diameter. Chopped-strand-mat is the randomly scattered fibres with a density of “450 g/m^2^” and a fibre length of 12 to 24 millimetres. Meanwhile, WR is a bidirectional fabric made by interweaving direct roving with a density of “800 g/m^2^”. The E-glass’s tensile strength and modulus of elasticity are “3.3 to 3.5 GPa” and “80 to 81 GPa”.

### 2.2. Preparation of Composites

**Figure 1:**
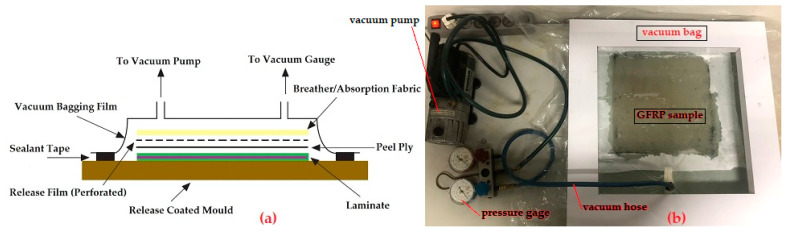
(**a**) The working principle of the vacuum technique; (**b**) A sample of GFRP being vacuumed.

Table 1 shows six types of GFRP composite samples that were designed to be three millimetres thick. The first way to make the samples was by hand lay-up, followed by vacuuming in a vacuum bag at room temperature. Vacuuming of material samples aims to control the number of voids. Figure 1 depicts the working principle and equipment of the vacuum bag we use. The composite consisted of three reinforcing fibre layers, namely, CSM450, WR800, and CSM450. The constituent materials, such as Ortho-UP, MEKP, ATH, CC, CSM450, and WR800, were taken from storage at room temperature to emulate the same conditions as the actual manufacturing of gutters at the factory.

The ortho-UP and fillers were mixed using a 3000-rpm rotary stirrer for five minutes, during which the air bubbles in the matrix will naturally disappear. Next, MEKP was added to the matrix and stirred gently using a stick. The composite sample work was carried out starting from the first layer. The CSM450 was doused with a matrix and then rolled with a steel roller to flatten it and to remove the air bubbles. The same process was repeated for the second layer of WR800 and the last layer of CSM450. The sample was then put into a vacuum bag and vacuumed at room temperature. After twenty-four hours, the samples were removed from the mould. They were cut into pieces to be used as specimens for testing and observation.

### 2.3. Physical and Chemical Properties

One physical property to be examined was the density of the GFRP composite sample, which was measured in accordance with the ASTM D792 standard [30]. The dimensions of the standard specimen were 30 mm × 30 mm × 3 mm. Five specimens were tested, and the mean value was reported. The density of a material is defined as mass per unit volume, while specific gravity is the ratio of a given material volume at “23 °C” to the same volume of deionised water. The specific gravity is measured by weighing both the specimen in air and the specimen when immersed in distilled water at “23 °C” using a weight/sinker and wire to hold the specimen to be fully submerged as required. Specific gravity and density are calculated in Equations (1) and (2), respectively.

(1) $$\mathrm{Specific}\;\mathrm{gravity}=\frac{a}{\left[\left(a+w\right)-b\right]}$$

Density (kg/m^3^) = specific gravity × 997.6 (2)

In (1), a is the actual mass of the specimen, without sinker and wire, in the air, b is the apparent mass of specimen (and of sinker and wire, if used) completely immersed and that of the wire partially immersed in liquid, and w is the apparent mass of the totally immersed sinker (if used) and that of the partially immersed wire. In the present study, because the density of the GFRP composite is greater than the density of water, no sinker and wire are needed; therefore, b is equal to a.

Another physical property to be measured was water absorption, which pertains to the amount of water absorbed by the GFRP composite specimen under certain conditions. The measurement of water absorption used specimens with dimensions of 76.2 mm × 25.4 mm × 3 mm, with the implementation of the test following the standard of ASTM D 570 [31]. Five specimens (except for samples UP100 and AT15: only four specimens, respectively) were tested, and the mean value was reported. The specimens underwent long-term immersion, and the specimens were weighed every 24 h for two weeks and then after every week until the weight gain was constant. Equation (3) describes the percentage of additional weight as a function of time (M_t_) due to water absorption. Equation (4) shows that M_t_ can also be expressed in terms of two parameters, namely, the diffusion coefficient or diffusivity (D) and the maximum moisture content (M_m_):

(3) $$M_{t}\;\left(\%\right)=\frac{W_{w}-W_{d}}{W_{d}}\times 100\%$$

(4) $$M_{t}=M_{m}\;[1-\frac{8}{\pi^{2}}\exp\;\{-\left(\frac{\mathrm{Dt}}{h^{2}}\right)\pi^{2}\}]$$

W_w_ and W_d_ are the weights of wet and dry materials, respectively. Maximum moisture content (M_m_) is the average of several consecutive measurements at the maximum value obtained after exposure within a particular time. The variables t and h are the time and thickness of the sample. Finally, D is determined using the initial linear part of the water absorption curve.

Concerning the chemical properties, the test to examine the fire-resistance properties of the composite material is based on the ASTM D635 [32]. It is the standard test method for the burning rate and the extent and time of burning plastics horizontally. The specimen of 125 mm × 13 mm × 3 mm was laid in a horizontal position with an inclination of 45 degrees to the horizontal plane at one end and then was ignited by fire from methane gas with a density of “37 MJ/m^3^” for 30 s. The burning rate was recorded for distances of the specimen from 25 to 100 mm. Tests on five specimens resulted in all samples’ average burning rate.

### 2.4. Mechanical Properties

The hardness of the GFRP composite samples was measured using a Barcol impressor following the ASTM D2583 standard [33]. The hardness reading scale is 0–100, which is used to assess whether the polymerisation of the UP resin is going well or not. For most FRP thermoset composites, the Barcol hardness scale will likely read between 35 and 45 once the resin matrix has wholly been polymerised. Barcol hardness testing/measurement at twenty different points for each sample resulted in an average Barcol hardness.

The flexural properties of GFRP composites were tested using the three-point bending method according to the ASTM D790 standard [34]. The dimensions of the standard test object were 133 mm × 12.7 mm × 3 mm. The test conditions were room temperature with a “5 mm/min” crosshead speed using a universal testing machine (Servopulser, Shimadzu with “20 kN” load cell). Equations (5) and (6) are the formula to calculate the flexural strength S (MPa) and the modulus of elasticity E_b_ (MPa), respectively. In this case, P (N) is the maximum load when the test object breaks, L (mm) is the distance between supports or spans, b (mm) is the width of the test object, d (mm) is the thickness of the test object, and m is the slope of the tangent to the initial straight line. Tests on five specimens (except for samples AT15 and AT30, only four specimens respectively) resulted in all samples’ average flexural strength and modulus of elasticity.

(5) $$S=\frac{3\;P\;L}{4{\;b\;d}^{2}}$$

(6) $$E_{b}=\frac{0.17{\;L}^{3}\;m}{{b\;d}^{3}\;}$$

To test the impact strength, the Charpy impact test was performed on a non-notched specimen based on the ASTM D6110 standard [35]. The dimensions of the specimen were 127 mm × 10 mm × 3 mm. Tests on five specimens resulted in all samples’ average impact strength.

### 2.5. Scanning Electron Microscopy

The microstructure of the fracture surface of the six samples of the GFRP composite from the flexural test was examined using scanning electron microscopy (SEM). Sputter coating with a thin palladium gold layer should be applied to the fracture surface, which was carried out in a vacuum for conductivity before the examination. These observations use the acceleration voltage of 10 kV at a working distance of 10 mm.

## 3. Results and Discussions

**Figure 2:**
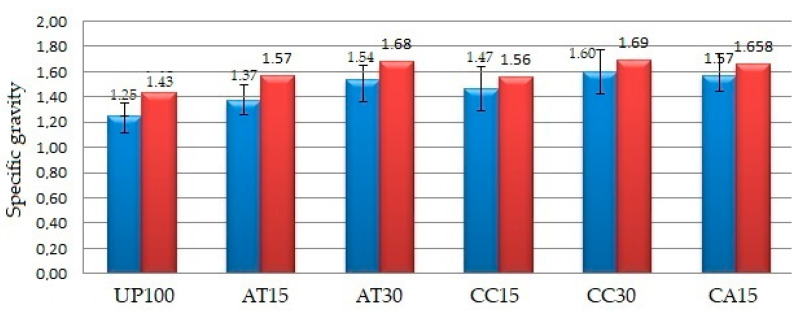
Specific gravity of the GFRP samples.

**Figure 3:**
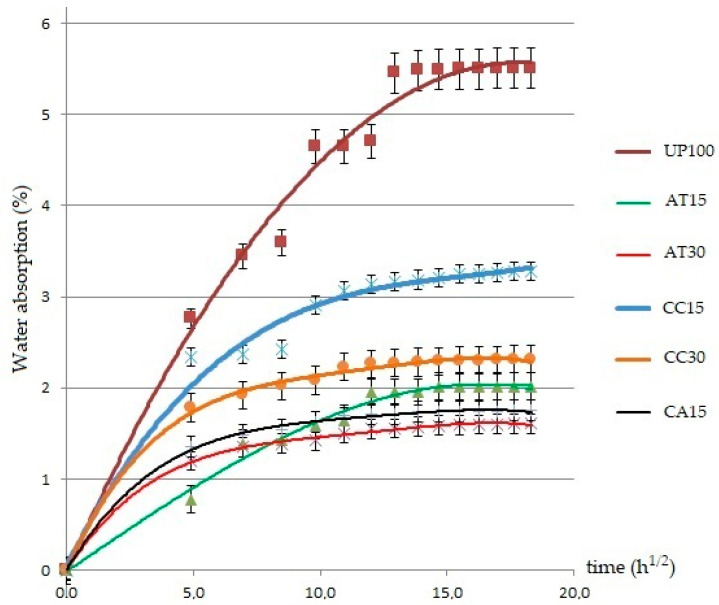
Water absorption curve of the GFRP samples.

**Figure 4:**
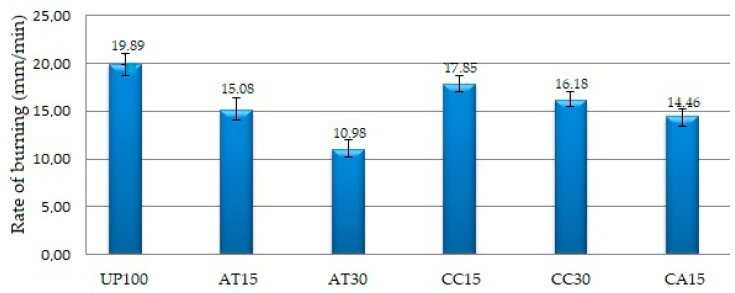
Rate of burning of the GFRP samples.

**Figure 5:**
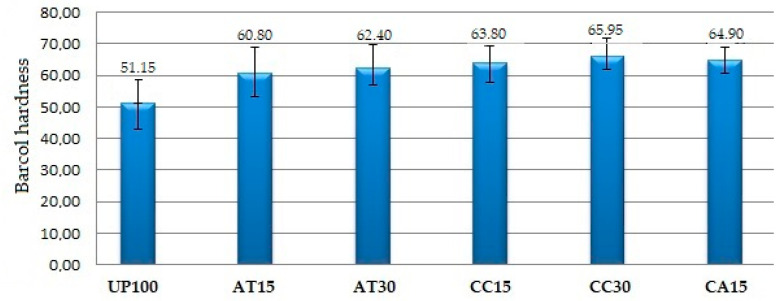
Barcol hardness of the GFRP samples.

**Figure 6:**
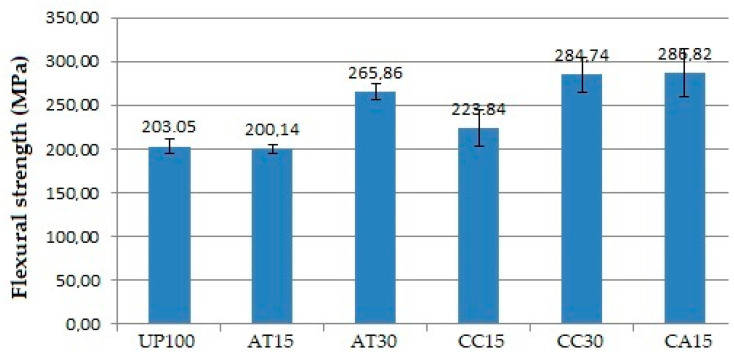
Flexural strength of the GFRP samples.

**Figure 7:**
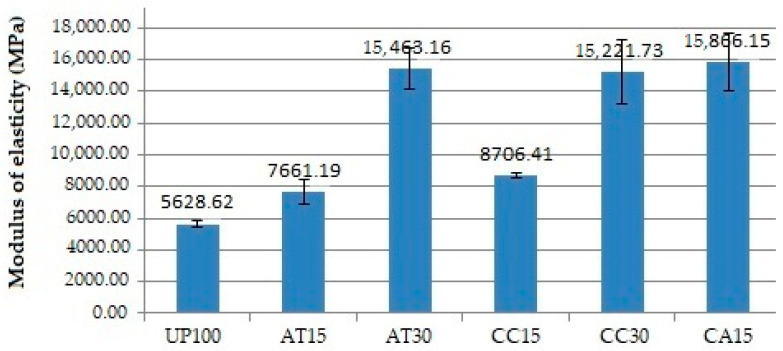
Modulus of elasticity of the GFRP samples.

**Figure 8:**
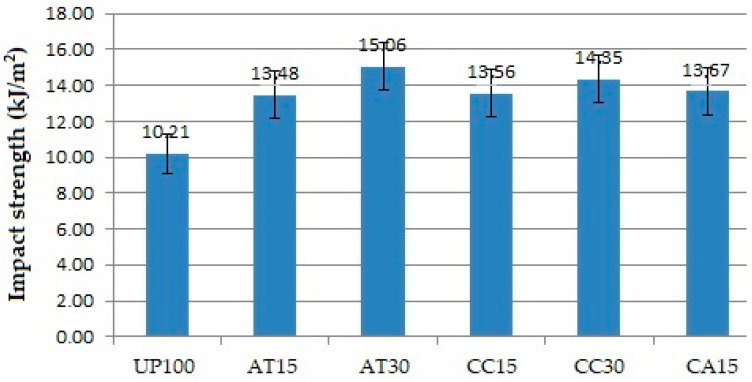
Impact strength of the GFRP samples.

**Figure 9:**
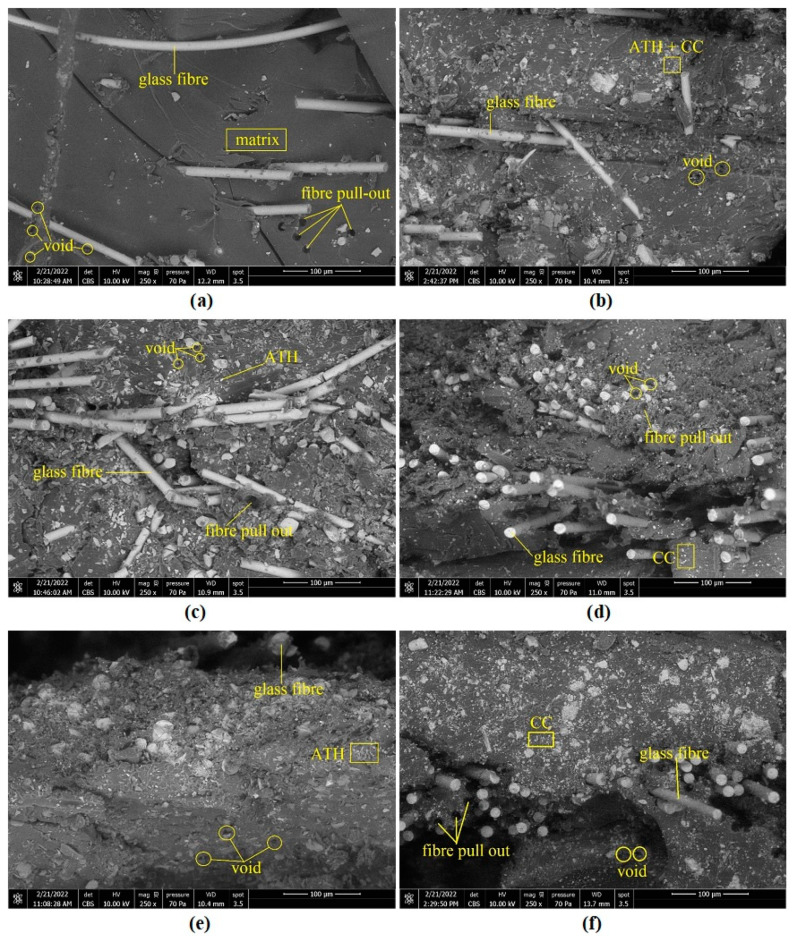
SEM micrographs of flexural fracture surfaces: (**a**) UP100, (**b**) CA15, (**c**) AT15, (**d**) CC15, (**e**) AT30, (**f**) CC30.

Table 2 shows the experimental results on the physical, chemical, and mechanical properties of the GFRP composite material samples. Further explanation of each property was given in graphical form as depicted in Figure 2, Figure 3, Figure 4, Figure 5, Figure 6, Figure 7 and Figure 8. Figure 9 shows the fracture morphology from the flexural test of all samples. 

### 3.1. Effect of Fillers on Specific Gravity

Figure 2 shows the effect of the addition of ATH and CC fillers into the UP resin on the specific gravity of all samples, as mentioned in Table 1. The graph on the left (blue) refers to experimental measurements according to the ASTM D792 standard, while the one on the right (red) represents the theoretical calculation. Theoretical calculations were obtained by considering each element’s weight and density fractions that made up the matrix. The specific gravity of the matrix elements, namely, UP, ATH, and CC, amounts to 1.11, 2.42, and 2.60, respectively. This theoretical calculation did not consider the presence of bubbles or voids when the samples were made; as a result, the specific gravity was higher than the experimental results. All samples of GFRP composite materials contained voids, causing the specific gravity value from the experimental measurements to be lower than the theoretical calculation. The density of composites containing CC filler was found to be higher than that of composites containing ATH. This observation was attributed to the CC filler density being higher than the ATH filler density. Even though vacuum bags were used, the voids in the samples were still present. While the vacuum bags reduced voids as a result of the hand lay-up process, the results of observing the fracture morphology of the samples clearly still showed the presence of voids in all samples. As depicted in Figure 2, the specific gravity values from the lowest to the highest were UP100 (1.25) < AT15 (1.37) < CC15 (1.47) < AT30 (1.54) < CA15 (1.57) < CC30 (1.60).

### 3.2. Effect of Fillers on Water Absorption

Figure 3 shows the relationship between the percentage of water absorption (M_t_) with the square root of the time (t^1/2^) of immersion of the GFRP composite samples in distilled water at room temperature.

The water absorption process for all specimens was linear at first, then slowed down and approached saturation point after a long time. The findings indicate that the water absorption behaviour of the composites accorded with Fick’s law. The all-composite samples showed different behaviour of water absorption. The composites sample without filler (UP100) was a composite with the highest percentage of water absorption at 5.51%, while the lowest was the composite containing 30 PHR of alumina trihydrate filler (AT30) at 1.61%. The diffusivity (D) and the maximum moisture content (M_m_) demonstrated the water absorption behaviour. As shown in Table 2, the highest value of M_m_ and D was in sample UP100, while the lowest was in sample AT30. Both M_m_ and D values decreased in the following order: AT30 < CA15 < AT15 < CC30 < CC15 < UP100.

The UP100 sample was a pure resin matrix composite without fillers, with a fibre weight percentage of 40 parts and a matrix of 60 parts. The percentage of fibre weight of the UP100 sample was the highest, 40 parts by weight, compared to other samples—CC15 and AT15 with 36 parts by weight and CC30, AT30, and CA15 with 33 parts by weight. These E-glass fibres carry air that contains moisture. The fibre is hydrophilic, meaning that it absorbs moisture from the atmosphere [25]. The highest fibre content is UP100 (40%), followed by AT15 and CC15 (36%) as well as AT30, CC30, and CA15 (33%). The more fibre content in the composite, the more air and water vapor content, which suggests that more voids are formed in the composite. The voids are still present even though the samples have been vacuumed to reduce them. These voids may in turn cause water absorption when the composite is immersed. The more voids, the higher the water absorption. The addition of fillers into the UP resin can then help reduce water absorption into the composite. In particular, ATH and CC fillers play an essential role in improving the composite properties against water absorption by adhering to the fibre surface in micro size and resultantly preventing the fibre from absorbing water [24]. The fibre that is originally hydrophilic will become hydrophobic. When compared, ATH filler is observed to be better than CC filler in making the composite absorb less water when the composite is immersed. In other words, ATH fillers are more hydrophobic than CC fillers.

The data show that loading of 15 and 30 PHR fillers accompanied by a decrease in the weight fraction of the fibre in the composite played a role in reducing water absorption in the composite. Composites containing ATH 30 PHR filler had the lowest water absorption, which correspond to the findings of other researchers who discovered that the addition of filler to the UP resin could decrease the water absorption properties of the composite [24,25,36]. Nevertheless, other researchers have cautioned that excessive filler loading can result in agglomeration, which produces more voids and leads to higher water absorption. In this study, the amount of filler at 30 PHR in the UP resin still provides a homogeneous distribution of filler particles in the composite matrix, as observed in the SEM microstructure image.

### 3.3. Effect of Fillers on Fire Resistance

Table 2 shows that the composite using pure resin without filler (UP100) had the highest rate of burning as per ASTM D635, which was “19.89 mm/min”, while the composite using alumina trihydrate 30 PHR (AT30) had the lowest, which was “10.98 mm/min”. As shown in Table 1, in general, the addition of fillers led to the reduction of the resin percentage in the composite. Therefore, as UP resin is flammable, the lower the resin percentage, the lower the rate of burning. Figure 4 depicts the effect of fillers on the fire resistance of the GFRP composite samples. The rate of burning in the ascending order was AT30 < CA15 < AT15 < CC30 < CC15 < UP100.

In addition to the amount of resin, the addition of ATH filler in UP resin decreases the burning rate of the composite, thereby increasing its fire resistance. ATH in a composite normally breaks down at an ambient temperature of around “180 °C” (“356 °F”) due to the exposure to excessive heat and subsequently releases moisture and water vapor [23,37,38]. As a consequence of the presence of H_2_O, thermal energy falls, causing a reduction in the composite combustion rate. In other words, as ATH in the composite functions to absorb heat, the higher the ATH content, the better the flame retardant properties. Specifically, adding ATH to the UP–resin composite can increase the limiting oxygen index, improve flame resistance, prolong ignition time, and reduce heat release rate. The ATH particle size also affects the fire resistance properties of the UP resin, meaning that the same mass ratio of submicron filler and nanofiller will provide better fire resistance. The two factors, the tiny amount of resin and the role of ATH as a fire retardant, explain why the AT30 composite has the lowest combustion rate. These findings are in line with those of other researchers who used ATH as a filler that functions as a fire retardant [23,37,39,40,41].

The findings have shown how ATH fillers outperform CC fillers in influencing the physical properties (e.g., density and water absorption) and chemical properties (e.g., fire resistance) of the composite. This is a technical advantage that gutter manufacturers must pay attention to in choosing the type of constituent material. One drawback of ATH fillers over CC fillers is that the former cost ten times more than the latter. However, because the price of ATH filler is about three and a half times of the price of UP resin and even the price of CC filler is thirty-five times cheaper than the price of UP resin, the use of fillers of 30 PHR can then replace the total weight of UP resin, potentially lowering the composite price. Whether the gutter manufacturers will choose ATH or CC filler, or a mixture of both as a UP resin filler, the present study has provided insightful considerations before the manufacturers make decisions.

### 3.4. Effect of Fillers on Hardness Properties

The Barcol hardness scale for GFRP composites indicates whether a composite has been fully cured. In this study, all samples have undergone sufficient curing or polymerisation because the hardness value is greater than 45, with the range of hardness values from 51.15 to 65.95 (see Table 2). Figure 5 depicts the relationship between the addition of filler and the hardness of the composite.

The addition of fillers ATH, CC, and a mixture of ATH and CC, ranging from 15 to 30 PHR, played a role in increasing the hardness of the composite. The hardness values in an ascending order were UP100 < AT15 < AT30 < CC15 < CA15 < CC30. In particular, the hardness of the sample filled with CC filler was higher than the sample filled with ATH filler, just as the hardness index of CC was higher than that of ATH. The CC and ATH fillers have Mohs scale hardness values of 3 and 2.5, respectively [42]. This suggests that the hardness of the composite sample derived is also related to the fillers used. These findings echo those of other researchers, who revealed that the addition of ATH or CC fillers into the UP resin up to 10 to 30 PHR increased the composite hardness value and reduced it when the filler was beyond 30 PHR [21,26]. The hardness values of all these material samples indicate that the fillers and UP resin mixture still provide a homogeneous matrix. The SEM images of all composite material samples show a homogeneous distribution of both filler and UP resin.

### 3.5. Effect of Fillers on Flexural Properties

Figure 6 and Figure 7 show the relationship between the addition of fillers and the flexural strength and modulus of elasticity of the GFRP composite samples. The addition of fillers such as ATH, CC, and a mixture of ATH and CC was found to play a role in increasing the flexural strength and modulus of elasticity of the composite materials. The lowest and highest flexural strength and modulus of elasticity were observed in UP100 and CA15, respectively.

The average flexural strength of the AT15, compared to the UP100, was slightly lower. However, the standard deviation of the AT15 data for the upper maximum deviation of the range gave a higher flexural strength value than the mean and a lower deviation of the UP100 flexural strength. Furthermore, the AT30 provided an even higher average flexural strength. These findings demonstrate how the addition of ATH filler tends to increase the flexural strength. The same results were observed with the CC fillers and the mixture of ATH and CC fillers, as well as the modulus of elasticity.

The addition of fillers of 15 PHR and 30 PHR, both ATH and CC, and a mixture of ATH and CC increased the flexural strength and modulus of elasticity of the GFRP composite, in line with the findings of earlier studies. The addition of filler up to 20–25% has been found to increase flexural strength, and a value greater than 25% begins to cause a decrease [21,22,25]. The higher the number of fillers, the higher the tendency of the filler to agglomerate. Voids may be formed as a result, causing a decrease in the fibre and matrix interface as well as the ability of the material to resist deformation and strain. The results finally confirm that the mixing of ATH, CC, and ATH and CC fillers into the resin still provides a homogeneous matrix, as evident from the SEM image observations.

It is known that fillers as a particle in the resin can serve as a barrier to micro-crack propagation from the composite matrix. As ATH and CC fillers are harder and stiffer than polymerised UP resins, micro-crack propagation can be hindered [21]. There will be a reduction in matrix deformation and strain, especially around the particles, namely, the resin–particle interface. The data from Table 2 and Figure 6 and Figure 7 illustrate that ATH and CC exhibited almost the same effect in increasing flexural strength and modulus of elasticity, with CA15 having the highest flexural strength and modulus of elasticity value of “286.82 MPa” and “15,866.15 MPa”, respectively. It is shown that mixing fillers up to 30 PHR into the UP resin still provides a relatively homogeneous distribution of filler particles. Overall, it is evident that all types of ATH and CC fillers increase flexural strength and modulus of elasticity [26].

### 3.6. Effect of Fillers on Impact Properties

Figure 8 shows that the composite matrix filled with ATH, CC, and a mixture of ATH and CC with as much as 15 PHR and 30 PHR had higher impact strength compared to composites with pure resin matrix without fillers. Furthermore, compared to CC filler, ATH filler exhibited higher or better impact strength. UP100 had the lowest impact strength. The impact strength of the CC15 was lower than that of the AT15, just as that of CC30 was lower than that of AT30. Overall, ATH fillers provide better technical properties than CC fillers.

The filler particles, which are homogeneously mixed with the resin, allow the matrix to have an excellent interfacial bond with the fibre. Filler as a particle in the resin serves as a barrier to micro-crack propagation from the composite matrix, thereby providing a strengthening effect on the impact strength. The higher the amount of filler in the resin, the more impact strength it has. ATH filler also turned out to outperform CC filler, as can be seen from the impact test results. The quality of the interfacial bond between the fibre and the matrix containing ATH was accordingly better than the matrix containing CC.

### 3.7. Scanning Electron Microscopy Studies

Figure 9 shows the SEM micrographs of the flexural fracture surface of GFRP composite samples loaded with ATH and CC fillers, respectively: (a) UP100, (b) CA15, (c) AT15, (d) CC15, (e) AT30, and (f) CC30. It is noticeable that the ATH and CC particles of 15 PHR at ATH15 and CC15 were uniformly distributed in the composite matrix. In composites with particles of 30 PHR, such as AT30, CC30, and CA15, the particle distribution was relatively even, although it started to look less homogeneous. The homogeneity of the particles in the composite matrix is influenced by good stirring quality. Good agitation ensures that the filler particles are uniformly dispersed and reduces the possibility of agglomeration caused by the addition of more filler particles. The fracture mode in all samples is a typical characteristic of brittle fracture.

The observed uniform dispersion resulted in better tensile modulus and flexural properties, as shown by fillers of 15 PHR at AT15 and CC15 compared to AT30, CC30, and CA15. Good dispersion can improve the composite strength due to mechanical interlocking, whereas poor dispersion may lead to agglomeration, where the polymer matrix is not distributed continuously and many particles are in direct contact with each other, resulting in poor bonding and adhesion at the interface. In all the composite samples, there were no clear clusters and gaps between the polymer matrix and the reinforcing fibre, indicating good dispersion and mixing of the resin matrix with the filler particles. It confirms the increase in the properties due to the addition of relatively well-dispersed ATH and CC particles, which consequently affect the mechanical properties. ATH and CC are rigid particles that are difficult to deform; therefore, the increase in ATH and CC content at 15 and 30 PHR in the UP resin in this study could lead to an increase in hardness, flexural strength, modulus of elasticity, and impact strength [21,23,36].

### 3.8. Overall Analysis

Observations have been made on the physical, chemical, and mechanical properties of the six samples of GFRP material, which represent guttering materials with a thickness of three millimetres. The specific gravity of the samples containing 30 PHR filler was almost the same, respectively, 1.54 for AT30, 1.57 for CA15, and 1.60 for CC30. The direct effect of specific gravity on the weight of the gutter is included as dead load. Based on these data, specific gravity is not a consideration in determining the selection of guttering material.

The second factor is the maximum moisture content, water in the composite causes weakening or reducing the mechanical properties of the composite. Water plays an important role in weakening the interfacial bond between the fibre and the matrix [25,27,28]. The test data show that the lowest maximum moisture content to the highest is AT30 (1.61%) < CA15 (1.75%) < AT15 (2.02%) < CC30 (2.31%) < CC15 (3.28%) < UP100 (5.51%). It is concluded that the best sample in terms of the lowest maximum moisture content is AT30, followed by CA15, AT15, and CC30. The low maximum moisture content needs to be considered when selecting gutter material.

The third factor is GFRP material response to fire, expressed by the burning rate as per ASTM D635. The flame propagation rate in the horizontal direction (mm/min) from the lowest to the highest, respectively, AT30 (10.98) < CA15 (12.20) < AT15 (13.51) < CC30 (15.56) < CC15 (15.99) < UP100 (19.89). It is concluded that the best sample in terms of the lowest rate of burning is AT30, followed by CA15, AT15, and CC30. The low rate of burning needs to be considered when selecting gutter materials.

The fourth factor is the hardness of the material. The Barcol scale hardness of the materials using the filler is almost the same, ranging from 60.80 to 65.95. These hardness values indicate that the resin polymerisation is complete. Hardness is not a determining factor in the selection of gutter material.

The fifth factor is the flexural characteristic which is represented by flexural strength and modulus of elasticity. The three best results for these parameters have almost the same value, namely, CC30, CA15, and AT30. Flexural characteristics need to be considered in the selection of guttering material.

The sixth factor is the impact strength of the material. The three best results for impact strength are AT30, CC30, and CA15. Impact strength needs to be considered in the selection of gutter material.

Technical considerations, as discussed above, have shown that the use of fillers is proven to improve physical, chemical, and mechanical properties, especially when compared to GFRP material without fillers. In particular, the material for gutters with a thickness of three millimetres from the six samples yielded the best three. They all use filler of 30 PHR, that is, AT30, followed by CA15, then CC30.

The last factor that deserves to be considered as completeness in materials selection for gutters is the price factor. As described in the previous description, the ATH and CC filler prices are three and a half times and thirty-five times cheaper than UP resin. The amount of resin to be replaced by the filler reaches thirty percent by weight or thirty PHR. The three samples starting from the CC30, CA15, and AT30 are worth considering in terms of material cost savings. The combination of technical and economic factors of material prices will be a careful consideration for gutter manufacturers to choose the suitable material according to consumer needs, whether AT30, CA15, or CC30.

## 4. Conclusions

The present study investigated E-glass/UP-resin composite samples that are filled with ATH and CC fillers. The composite thickness of three millimetres represented the thickness of typical commercial gutters. The composite consisted of two layers of E-glass CSM450 on the outside and one layer of E-glass WR800 as the middle layer. The six samples were compared for their physical, chemical, and mechanical properties: UP100 (composite with pure resin), AT15 and CC15 (composite with 15 PHR filler ATH and CC), AT30, CC30, and CA15 (composite with 30 PHR ATH fillers, 30 PHR CC fillers, and 15 PHR ATH fillers mixed with CC each). The physical and chemical properties being observed were density, water absorption, and fire resistance, while the mechanical properties being observed were hardness, flexural strength, modulus of elasticity, and impact strength.

In this study, the density, hardness, flexural strength, modulus of elasticity, and impact strength of the composite increased compared to those of the composites with pure resin. On the other hand, the maximum water absorption and burning rate in the horizontal direction decreased.

As a guttering material with a thickness of three millimetres, the AT30 sample has the best engineering properties, followed by CA15 and CC30. Regarding material cost savings, the CC30 sample is the cheapest, followed by the CA15 and the AT30. Determination of gutter material depends on consumer needs; the two considerations concerning the technical factor and the price factor will determine whether the gutter manufacturer chooses AT30 or CA15 or CC30 material.

## Figures and Tables

**Table 1:** The GFRP composite samples.

Sample Name	Reinforcement	Matrix			Weight Fraction (%)		Thickness (mm)
		Parts Per Hundredweight Resins (PHR)					
		Ortho-UP	ATH	CC	Reinforcement	Matrix	
UP100	CSM450 + WR800 + CSM450	100	0	0	40	60	3
AT15	CSM450 + WR800 + CSM450	100	15	0	36	64	3
AT30	CSM450 + WR800 + CSM450	100	30	0	33	67	3
CC15	CSM450 + WR800 + CSM450	100	0	15	36	64	3
CC30	CSM450 + WR800 + CSM450	100	0	30	33	67	3
CA15	CSM450 + WR800 + CSM450	100	15	15	33	67	3

**Table 2:** Physical, chemical, and mechanical properties of the GFRP samples.

Samples	Physical Properties				Chemical Properties	Mechanical Properties			
	Specific Gravity	Density (kg/m^3^)	Maximum Moisture Content (%)	Diffu- Sivity (×10^−12^ m^2^/s)	Rate of Burning (mm/min)	Barcol Hardness (1–100)	Flexural Strength (MPa)	Modulus of Elasti- city (MPa)	Impact Strength (kJ/m^2^)
UP100	1.25 ±0.12	1247 ±120	5.51 ±0.37	20.04 ±1.37	19.89 ±1.25	51.15 ±8.26	203.05 ±7.91	5628.62 ±212.61	10.21 ±1.09
AT15	1.37 ±0.11	1367 ±110	2.02 ±0.21	13.51 ±1.41	15.08 ±1.05	60.80 ±7.35	200.14 ±5.13	7661.19 ±743.14	13.48 ±1.30
AT30	1.54 ±0.15	1536 ±150	1.61 ±0.13	11.37 ±0.92	10.98 ±0.80	62.40 ±5.60	265.86 ±9.24	15,463.16 ±1321.84	15.06 ±1.33
CC15	1.47 ±0.14	1467 ±140	3.28 ±0.31	15.99 ±1.51	17.85 ±0.85	63.80 ±5.93	223.84 ±20.88	8706.41 ±164.92	13.56 ±1.35
CC30	1.60 ±0.16	1596 ±160	2.31 ±0.21	15.56 ±1.42	16.18 ±0.75	65.95 ±3.99	284.74 ±19.80	15,221.73 ±2034.79	14.35 ±1.34
CA15	1.57 0.12	1566 ±120	1.75 ±0.08	12.20 ±0.56	14.46 ±1.00	64.90 ±4.43	286.82 ±27.14	15,866.15 ±1786.81	13.67 ±1.33

## Data Availability

The data presented in this study are available on request from the corresponding author.
